# Heavy lipids protect against heavy metals

**DOI:** 10.18632/aging.204143

**Published:** 2022-06-22

**Authors:** Joshua L. Dunaief

**Affiliations:** 1 FM Kirby Center for Molecular Ophthalmology, Scheie Eye Institute, Perelman School of Medicine, University of Pennsylvania, Philadelphia, PA 19104, USA

**Keywords:** macular degeneration, AMD, oxidative stress, anti-oxidant, drug

Oxidative stress exacerbates age-related diseases. One of the most potent generators of oxidative stress is iron. This is especially problematic in the elderly, as iron accumulates in tissues with age. It is absorbed by the gut throughout the lifespan, but is not excreted. Several lines of evidence suggest it promotes aging [1]. Further, age-related inflammation can activate the cellular iron sequestration response (CISR) [2], a maladaptive application of a mechanism designed to deprive extracellular pathogens of the iron they need to grow. The CISR exacerbated neurodegeneration in an alpha-synuclein induced model of Parkinson’s and an iron chelator partially protected against neurodegeneration. This is consistent with a prior publication showing iron chelation is protective in the MPTP Parkinson’s model [3]. The CISR can also cause retinal pigment epithelial cell iron accumulation in mice with systemic inflammation.

Iron accumulation has been found in tissue from patients with Parkinson’s, Alzheimer’s, and age-related macular generation (AMD). In AMD, which is associated with systemic inflammation, the iron accumulation is especially prominent in the retinal pigment epithelium [4]. Several mouse lines with acute or chronic iron toxicity model aspects of these neurodegenerative diseases. Recently, we described a model with features of the geographic atrophy form of AMD [5]. In this model, iron is injected into the center of the mouse eye (intravitreal injection) causing acute oxidative stress and death of photoreceptors. Over the ensuing months, patches of retinal pigment epithelial death expand and coalesce, a feature in common with human geographic atrophy. In addition, intravitreal iron injection in one eye initiates an immune reaction against the contralateral eye, reminiscent of the human disease sympathetic ophthalmia.

To test whether retinal iron toxicity is primarily mediated by lipid peroxidation, and to evaluate a potential therapy for AMD, we studied an oxidation-resistant lipid. The most abundant lipid in photoreceptors is docosahexanoic acid (DHA), a polyunsaturated fatty acid (PUFA). A modified form of DHA, with deuterium substituted for hydrogens, is resistant to lipid peroxidation and can prevent lipid peroxidation chain reactions [6]. Deuterium, a non-radioactive isotope of hydrogen, is biocompatible and has been used safely in patients for metabolic imaging. We fed mice a diet containing either deuterated DHA (D-DHA) or a control diet that was identical except that DHA was not deuterated. The D-DHA fed mice were completely protected against the extensive photoreceptor and retinal pigment epithelial death caused by intravitreal iron injection in mice fed the control diet [7]. Complete protection was observed when the retinal D-DHA to DHA ratio was at least 1:1. No adverse effects of D-DHA were observed on the structure or function of the mouse retina when the D-DHA/DHA ratio was greater than 10:1 over a period of at least six months. Preliminary studies also show that D-DHA can halt retinal degeneration in models of retinal iron toxicity that progress slowly over many months.

Because oxidative stress and, specifically, DHA oxidation [8] have been implicated in AMD pathogenesis, a clinical trial of D-DHA, initially for the geographic atrophy form of AMD, seems warranted. D-PUFAs could also be tested in other age-related disease that feature lipid peroxidation.
